# Myxoid liposarcoma of right popliteal fossa: Case report and review

**DOI:** 10.1097/MD.0000000000047225

**Published:** 2026-01-16

**Authors:** Jialie Xu, Aohui Yan, Pengfei Yu, Kun Zhou, Zhengmao Zhang

**Affiliations:** a Department of Orthopedics, Yuhuan People’s Hospital, the First Affiliated Hospital of Wenzhou Medical University, Yuhuan, Zhejiang, China; b Department of Orthopedics, the First Affiliated Hospital of Wenzhou Medical University, Wenzhou, Zhejiang, China.

**Keywords:** MRI, myxoid liposarcoma, popliteal fossa, surgery

## Abstract

**Rationale:**

Myxoid liposarcoma (MLS) accounts for 30% of liposarcomas and typically arises in the thigh, but rarely arises in the popliteal fossa. Managing giant, neurovascular-abutting MLS in young adults is poorly characterized.

**Patient concerns:**

A 35-year-old man presented with a right popliteal mass growing over > 6 years, accompanied by progressive swelling and incomplete flexion of the right knee. He denied smoking, denied alcohol use, and had no family history of malignancy.

**Diagnoses:**

magnetic resonance imaging showed a 179 × 154 × 99 mm multilocular cystic–solid lesion abutting/partly encasing the popliteal vessels and contacting the sciatic/common peroneal nerves. Preoperative cytology suggested benignity; definitive resection revealed MLS grade II with 20% round cell component (R1 margin adjacent to the vessel plane).

**Interventions:**

En bloc resection with function-preserving R1 margins was performed given neurovascular involvement; popliteal artery/vein and nerves were preserved. Postoperative external-beam radiotherapy was delivered to 60 Gy in 30 fractions (2 Gy/fraction).

**Outcomes:**

At the 18-month follow-up, progressive recovery of knee joint range of motion was observed, and no local recurrence or metastasis at 18 months. Magnetic resonance imaging at 6 and 18 months showed no local-recurrence or metastasis.

**Lessons:**

This case demonstrates that in young patients with popliteal MLS closely related to neurovascular structures, function-preserving R1 resection plus postoperative 60 Gy can achieve local-control while maintaining limb function, provided meticulous technique and guideline-concordant surveillance.

## 1. Introduction

Liposarcoma is a malignant tumor of adipocyte differentiation, of which myxoid liposarcoma (MLS) is a subtype, accounting for about 30% of liposarcomas. MLS usually occurs in the proximal extremities, and it is reported in the literature that about 2-thirds of cases originate in the thigh.^[1]^ Popliteal fossa involvement is uncommon and may present late because of painless enlargement and deep location. Since MLS is generally a painless growth mass and often has no obvious symptoms, the mass is usually large when patients come to the hospital, with a median of about 12cm.^[2]^ The average age of the MLS patients is about 50 years old, and there are few reports on the treatment of young patients, especially reports on the lesions in the popliteal fossa are rarely reported. The etiology of MLS remains controversial, and the descriptions in the literature are mainly at the genetic level. More than 90% of MLS are genetically characterized by a t(12:16)(q13;p11) translocation, which results in the expression of the FUS-DDIT3 fusion protein, and changes in round cell content are characteristic, which is a poor prognostic factor for it (> 5%).^[3,4]^ Therefore, MLS can be divided into high-grade and low-grade according to the number of round cells.^[5]^ Local relapses occur in about 9% to 12% with distant metastases in about 9% to 33% of cases, and it usually metastasizes to atypical areas such as the abdomen, bones, lungs or contralateral limbs.^[3,6,7]^ This article introduces the treatment process of a young patient and reviews relevant literature, hoping to deepen our understanding of MLS in the popliteal fossa and provide a reference for our follow-up treatment of related cases.

## 2. Case report

**Figure 1.**
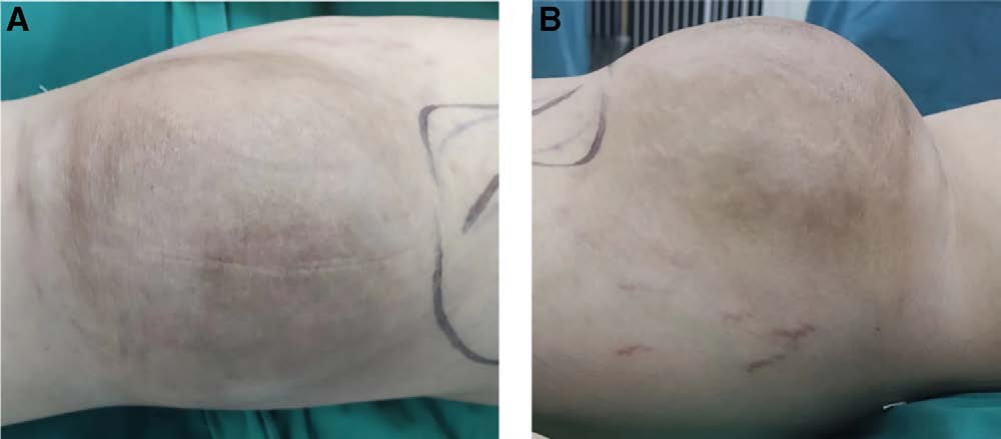
(A, B) Preoperative right popliteal fossa mass with dilated skin veins and no ulceration.

**Figure 2.**
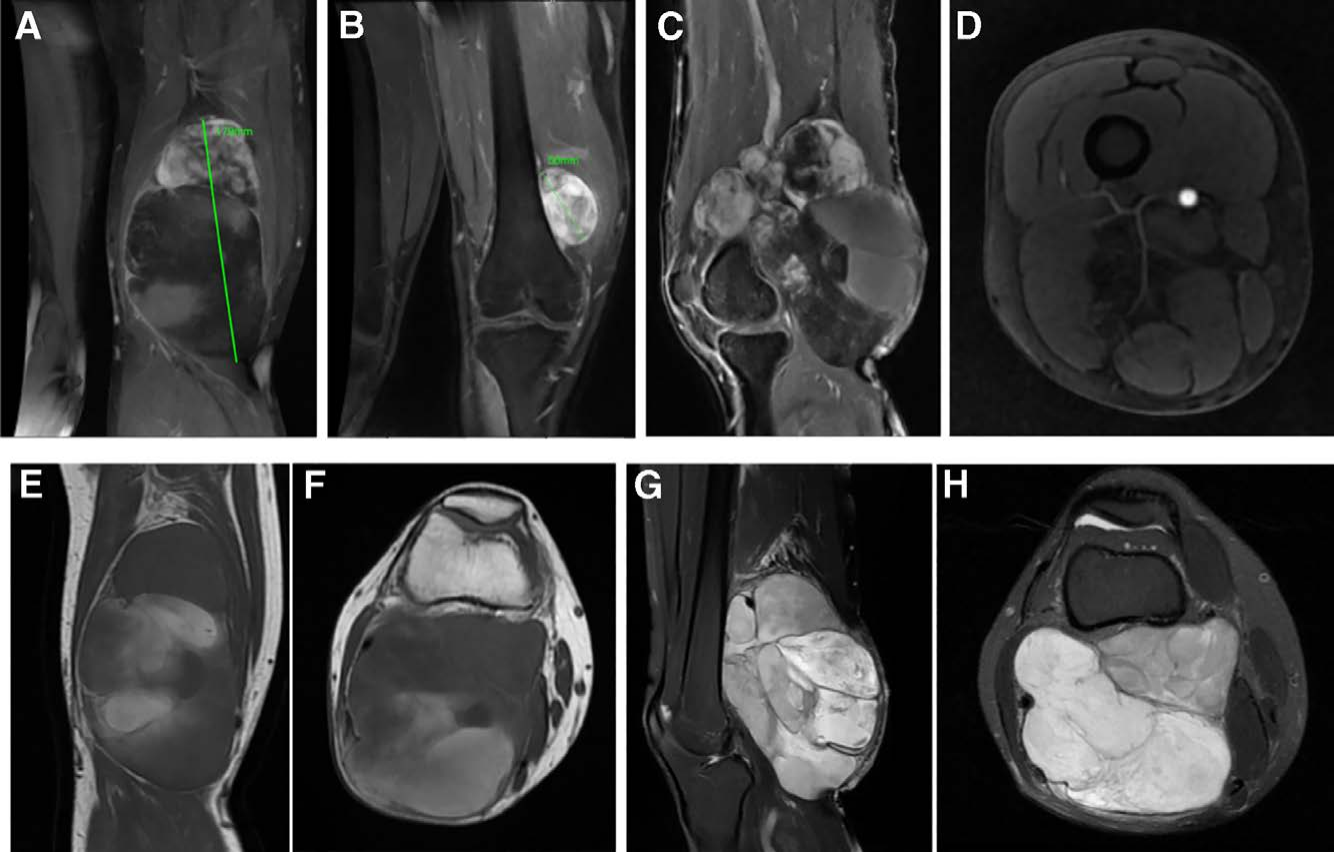
Preoperative MRI images. (A-B) Coronal view of enhanced MRI scan shows a giant mass. (C) Sagittal view of enhanced MRI scan shows that the mass consists of 2 components. (D) Horizontal view of enhanced MRI scan shows the mass is highly vascularized. (E) Coronal view of TIWI of MRI scan shows multilocular cystic changes inside. (F) Horizontal position of TIWI of MRI scan shows high and low mixed signals. (G) Sagittal view of T2WI of MRI scan shows partial non-enhancement of the mass. (H) Horizontal position of T2WI of MRI scan shows The multilocular cystic–solid mass encases the popliteal vessels with septal enhancement.

The patient, male, 35 years old, was in good health and had no history of smoking, alcohol consumption, or familial malignancy. He came to our outpatient clinic on March 11, 2022, due to “discovery of a mass in the right popliteal fossa for more than 6 years,” and planned to be admitted to the hospital for “malignant tumor of the right knee.” On physical examination, the right knee inability to fully flex, while extension remained preserved, a mass of about 20 × 12 cm in size can be palpated in his right popliteal fossa, and a mass of about 5 × 3 cm in size can be palpated on the inner side of the right knee, with clear borders, immovable, distended veins on the local skin surface, no skin ulceration, and the blood supply and feel of the extremities unobstructed (Fig. 1). Magnetic resonance imaging (MRI) of his right popliteal fossa was requested and showed increased soft tissue. MRI showed that a huge cystic and solid mass was seen in the right popliteal fossa, with high and low mixed signals on T1-weighted images, and the largest cross-sectional size was about 179 × 154 × 99 mm, with multilocular cystic changes inside, lobulated surface and clear borders. T2-weighted images showed inhomogeneous hyperintensity. And in contrast-enhanced scan, the solid part of the upper part of the mass showed continuous uneven patch and flocculent-like obvious enhancement, with no obvious enhancement in the multilocular cystic part of the middle and lower part of the mass, and the intracapsular septum was slightly enhanced. It showed that part of the mass protruded behind the right vastus medialis muscle, and the right femoral artery and popliteal artery were embedded, with significant outward offset compression and thinning (Fig. 2). He was also ordered to do a cytology biopsy, suggesting that benign lesions were considered. Meaning while, Single-Photon Emission Computed Tomography/CT showed no obvious bone metastases. At that time, it was suspected that the diagnosis could be liposarcoma.

**Figure 3.**
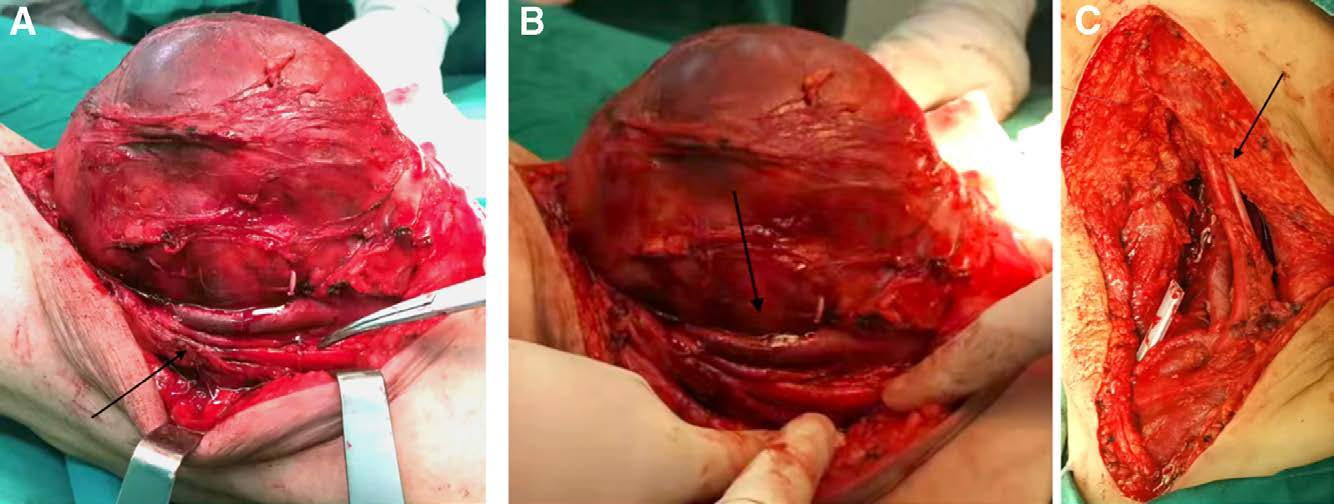
(A, B) During tumor dissection, part of the popliteal artery and vein traversed the mass; the tumor was large, and the vessels were carefully separated. The parts indicated by the arrows are from top to bottom: popliteal vein, popliteal artery, and sciatic nerve. (C) After tumor resection, residual tissue around the popliteal vessels was completely removed. The arrow indicates intact popliteal artery, vein, and nerve without injury. The parts indicated by the arrows are from left to right: popliteal vein, popliteal artery, and common peroneal nerve.

**Figure 4.**
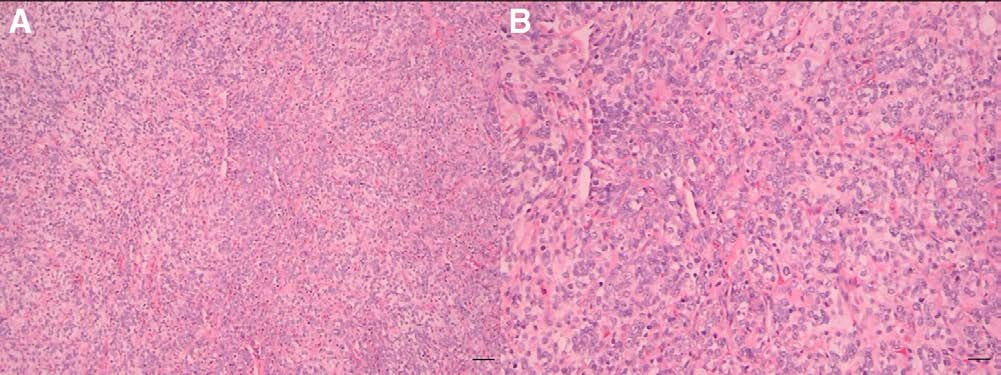
Postoperative pathological findings suggest myxoid liposarcoma (grade II (small round cells account for about 20% of the tumor)). (A) HE 100X. (B) HE 200X. HE = hematoxylin–eosin.

**Figure 5.**
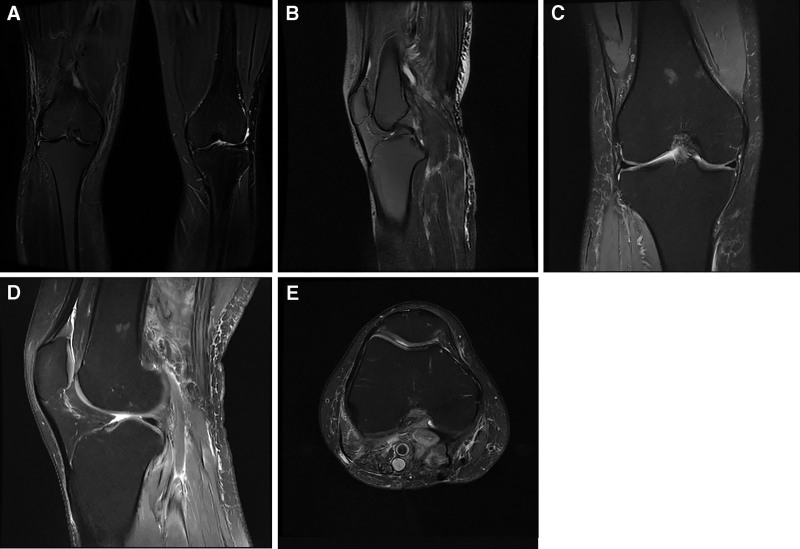
Postoperative MRI. (A–B) 6-month postoperative MRI of the right popliteal fossa showing decreased signal intensity in the popliteal fossa soft tissues; scattered patchy and cord-like areas of low signal on T1WI and high signal on T2WI within soft tissue and subcutaneous tissue, with partial cord-like enhancement. No abnormal signal was noted in the right knee joint bone. (C–E) ≥18-month MRI again showed no local recurrence at the operative bed. MRI = magnetic resonance imaging, T1WI = T1-weighted images, T2WI = T2-weighted images.

Based on the above judgments, surgical treatment was arranged after communication with the patient. During the operation, the capsule of the mass was intact, and the local blood supply was abundant. The tumor size was about 20 × 17 ×10 cm, and a 5 × 7 cm mass was seen in the deep inner layer of the popliteal fossa. The tumor adhered to the sciatic nerve, popliteal artery, and popliteal vein and squeezed them to the outside, and the popliteal artery and vein partially penetrated the tumor. Due to the tumor’s extensive adhesion to surrounding structures, partial resection of adjacent muscles (semimembranosus, biceps femoris, and medial and lateral heads of gastrocnemius) was necessary. The rationale for muscle excision is 2-fold: to achieve an oncologically safe margin by including potentially infiltrated muscular tissue, and to reduce the risk of microscopic residual disease in the setting of an R1 resection margin adjacent to neurovascular bundles. The tumor was completely resected, and the popliteal artery and vein, sciatic nerve, and common peroneal nerve were preserved intact (Fig. 3). The mass was sent to intraoperative frozen pathology and routine pathological examination. The intraoperative frozen pathology showed that the mass was a myxoid spindle cell tumor with necrosis. Then we removed the semimembranosus, the biceps femoris, and the medial and lateral head of the gastrocnemius muscle to the normal limit of 5mm. After excision of the tumor, we rinsed repeatedly with distilled water, diluted iodophor, and normal saline, followed by hemostasis, sutures, and bandaging. Postoperative paraffin pathology and immunohistochemistry results showed it was MLS (Ⅱ, small round cells make up about 20% of tumors) with CD34(+), CDK4(+), CK (-), Desmin (-), EMA (+), MDM (+), S-100 (+) and SMA (-) (Fig. 4). Our final diagnosis was: MLS. The patient recovered well after the operation and received radiotherapy (30 × 2 Gy) to the tumor bed with appropriate margins in the radiotherapy department of our hospital after discharge, consistent with postoperative dosing recommendations (60–66 Gy) for deep large STS and/or positive margins^[8]^. The patient underwent standardized postoperative surveillance. At 6 months after surgery, postoperative MRI of the right popliteal fossa demonstrated no residual or recurrent mass at the operative bed, with only expected postoperative changes. Over 18 months, surveillance MRI again showed no local recurrence (Fig. 5). Up to now, his knee joint function has recovered well, and there is no obvious sign of recurrence.

## 3. Discussion

Liposarcoma is a rare lipoblastoma, mesenchymal tumor, often involving deep soft tissues such as the extremities, esophagus, and retroperitoneum. There are 4 subtypes of liposarcoma: well-differentiated liposarcoma, dedifferentiated liposarcoma, MLS, and pleomorphic liposarcoma. MLS is the second most common subtype of liposarcoma, accounting for 30% of all liposarcoma cases and 10% of all soft tissue sarcomas.^[9–11]^ MLS mainly occurs in deep soft tissues of the extremities (most frequently the thigh) in an average 50-year-old patient, and it affects men more than women.^[12–14]^ Although it usually occurs in the lower extremities, we searched the databases of PUBMED, WANFANG, and CNKI with the keywords “knee or popliteal fossa” and “liposarcoma,” and found that only Mottahedi^[15]^ and Filho^[16]^ have carried out some studies in the past 10 years. The reported patients were 50 years old and 40 years old, and they were treated with surgery and radiotherapy. In addition, only some articles briefly mentioned the popliteal fossa MLS. In the case reported in this article, the patient is a 35-year-old male patient and the lesion, 20 cm, is located in the right popliteal fossa, which is relatively rare and has high reference value.

The preoperative diagnosis of MLS mainly relies on MRI and pathological examination. Preoperative MRI not only facilitates tumor diagnosis, but also provides surgeons with its relationship to peripheral nerve and vessels. MLS is mainly manifested as a multilocular mass on MRI, mixed type on T1-weighted images, in which the high signal is the fat component and the mucus component is low signal. T2-weighted images show high signal, and flocculent and lace-like high signal intervals can be seen at the same time. And edges and septa appear as mild to moderate enhancement on contrast-enhanced scans.^[17–20]^ At the same time, preoperative cytological biopsy is also helpful for diagnosis, but sometimes the results are not ideal due to various reasons, as in our results. The MRI in this case was similar to that reported in other relevant literature. The preoperative biopsy was considered a benign tumor, but combined with the imaging examination and the clinical manifestations of the patient, we still diagnosed liposarcoma preoperatively. We believe that the inconsistency between the pathological results of biopsy and the final surgical specimens may be caused by less tissue samples, necrotic sites, or the need for higher detection technology. FNAC/cytology of soft-tissue tumors has lower accuracy than core-needle biopsy, with false negatives driven by sampling error and necrosis, especially in large myxoid lesions where noncellular pools predominate. Image-guided CNB targeting the enhancing solid portions on MRI improves histologic yield and grading concordance.^[21]^ The final diagnosis of MLS relies on pathological examination of intraoperative specimens. Our benign cytology likely sampled cystic/necrotic components; definitive resection revealed MLS with ~20% round-cell component.^[22]^ Histologically, MSL is nodular, with a prominent myxoid matrix, numerous branched capillaries, and characteristic “chicken-wire” vasculature. There is no obvious pleomorphism in the nucleus, and round cells can be seen in some MSL, of which round cells > 5% are defined as high-grade MSL.^[23,24]^ In this case, round cells accounted for about 20%, which belonged to high-grade MSL. High-grade disease correlates with worse outcomes; our case (~20%) justified aggressive local therapy and close imaging follow-up.^[5]^

In terms of treatment, surgery is the method of choice for MLS. Surgical resection is divided into R0, R1, and R2. Among them, R0 is achieved by removing the lesion, the surrounding pseudocapsule or normal tissue, and the normal tissue resection range is 1~2 cm. The resection method of R1 is the same as that of R0, but through the capsule during resection, which may cause some tumor cells to remain on the resection margin. In R2, the tumor cannot be completely removed because the tumor is attached to blood vessels or nerves, or for other reasons.^[10]^ Resection margins affect the postoperative recurrence rate of patients. This view is also supported by the study by Dürr HR^[3]^, who showed that the local recurrence rate was 0 after R0 resection and 33% after R1 resection. At the same time, the study by Zheng K.^[4]^ showed that the local recurrence rate of patients with wide resection was lower than that of patients with marginal resection, but there was no significant difference in survival analysis between them. Although marginal resection increases the risk of recurrence, it is beneficial to patients with limb preservation and does not increase the risk of death, so local marginal resection can be considered first in surgical treatment. Wide/R0 margins achieve the lowest local-recurrence risk; however, in neurovascular-adjacent MLS, marginal/R1 resection can be acceptable for limb preservation when combined with adjuvant RT, without compromising survival.^[4]^ In the case we reported, the tumor invaded the popliteal artery and vein, and was close to the nerve. The blood vessels and nerves were all compressed to the outside. After discussion in the department before surgery, cytology-only sampling of myxoid-dominant, multilocular lesions is prone to underdiagnosis because hypocellular pools and cystic change yield scant lesional cells; targeting enhancing solid septa with core-needle biopsy reduces false-negatives. In this case, R0 resection was anatomically prohibitive without vascular/nerve sacrifice; thus, an R1 functional margin was accepted with planned adjuvant 60 Gy radiotherapy, to preserve the limbs and function of the patient to the greatest extent, we performed local resection. The tumor was completely removed, and the popliteal artery and vein, and nerve were preserved intact. Leveraging the recognized radiosensitivity of MLS and documented local-control benefit in close/positive margins. This strategy preserved limb function while maintaining disease control at ~18 months.

At present, radiotherapy and chemotherapy are also widely used in MLS. Compared with other soft tissue sarcomas, MLS is more sensitive to radiotherapy and chemotherapy.^[23]^ In a study of 23 patients who received preoperative radiotherapy, Salduz^[25]^ found that only 1 case developed local metastases. Other related literatures also have similar results: perioperative radiotherapy can significantly reduce the local recurrence rate of postoperative tumors.^[26–28]^ At present, it is still controversial whether neoadjuvant radiotherapy or adjuvant radiotherapy is beneficial to the survival rate, but the literature has confirmed that they have the same effect in preventing local recurrence.^[29]^ There are also reports in the literature that preoperative radiotherapy has a lower local recurrence rate than postoperative radiotherapy.^[30]^ However, preoperative radiotherapy has a higher risk of related surgical complications, such as hematoma, infection, and wound rupture, etc^[2]^ For postoperative RT, 60 to 66 Gy in 1.8 to 2 Gy fractions is widely recommended; we delivered 60 Gy, balancing efficacy and toxicity. Randomized data show pre-op 50 Gy has a higher wound-complication risk versus post-op RT, while both achieve excellent local control; in our large, vessel-involving tumor with R1 margin, postoperative RT was selected to avoid heightened wound morbidity.^[8,31]^ The patient reported in this article received radiotherapy after surgery, and the wound recovered well. Now, the patient has no obvious signs of recurrence.

In terms of chemotherapy, there are also a variety of drugs that have proven effective, such as ifosfamide, epirubicin, and trabectedin, etc^[32,33]^. A recent clinical trial by Gronchi^[34]^ showed that trabectedin has a significant effect on MLS. However, there are few reports on the pros and cons of neoadjuvant chemotherapy and adjuvant chemotherapy, and further research is needed to confirm.

## 4. Conclusion

This rare case of a giant MLS in the popliteal fossa demonstrates that, in young patients with popliteal MLS abutting neurovascular structures, when R0 resection is anatomically prohibitive, complete surgical resection with preservation of major neurovascular structures can be achieved in function-preserving R1 resection plus adjuvant 60 Gy radiotherapy, resulting in good postoperative functional recovery and no recurrence during follow-up. MRI and fine needle aspiration cytology are valuable for preoperative evaluation, while surgery remains the mainstay of treatment. Adjuvant radiotherapy may further reduce the risk of recurrence. This article aims to improve the understanding of popliteal MLS.

## Author contributions

**Conceptualization:** Jialie Xu.

**Writing – review & editing:** Aohui Yan.

**Validation:** Pengfei Yu.

**Methodology:** Kun Zhou, Zhengmao Zhang.
